# Promoting Teachers’ Organizational Commitment: The Effects of Authentic Leadership, Teachers’ Well-Being and Social–Emotional Competence

**DOI:** 10.3390/bs14100862

**Published:** 2024-09-24

**Authors:** Zeqing Xu, Nicholas Sun-Keung Pang

**Affiliations:** 1 Department of Education Management, Faculty of Education, East China Normal University, Shanghai 200062, China; 52264110004@stu.ecnu.edu.cn; 2 Department of Education, Faculty of Education, East China Normal University, Shanghai 200062, China

**Keywords:** authentic leadership, organizational commitment, teacher well-being, social–emotional competence, moderated mediation

## Abstract

Committed and satisfied teachers have been increasingly recognized as valuable assets in enhancing the effectiveness of schools and adapting to evolving education reforms. This study investigates how and under what conditions principals’ authentic leadership contributes to teachers’ organizational commitment. Valid data collected from 476 teachers in seven primary schools in mainland China were analyzed using structural equation modeling (SEM), regression analysis and bootstrapping tests. The results yielded a partial mediation model, finding a significant direct effect of principals’ authentic leadership on teachers’ organizational commitment and an indirect effect through the mediating role of teachers’ well-being. Moreover, teachers’ social–emotional competence positively moderated the relationship between principals’ authentic leadership and teachers’ organizational commitment. This study underscores the theoretical importance of teachers’ affective pathways and the boundary condition linking principals’ authentic leadership to teachers’ organizational commitment, while it also offers practical recommendations for school leaders.

## 1. Introduction

In today’s competitive and ever-changing landscape, committed employees play a major positive role in any organization [1]. Similarly, in order to adapt to large-scale and evolving education reforms around the world, stable and committed teachers have been increasingly recognized as the most valuable assets. However, in recent years, increasing teacher turnover rates have become a growing concern globally. Teachers’ organizational commitment is a psychological link between teachers and schools, which is negatively related to teachers’ turnover intention [2]. The significance of teachers’ organizational commitment in promoting teachers’ team development and student performance has also been extensively recognized in various educational contexts [3,4].

### 1.1. The Teaching Profession in China

With a teaching workforce of approximately 5.8 million in primary schools [5], China is also facing extremely high rates of attrition among teachers and a subsequent shortage of high-quality teachers. National research indicated that nearly 62% of full-time primary and secondary school teachers have a strong intention to leave the teaching profession in China [6]. To solve this problem, policymakers in China have implemented efforts to ensure teachers’ well-being, personal accomplishment and social status [7], which could increase teachers’ commitment to the teaching profession. However, evolving education reforms and China’s cultural context have placed specific demands on most teachers and contributed to shaping a low-level organizational commitment profile in some schools. Related research has found that teachers are experiencing more and more mental problems, such as low job satisfaction, emotional exhaustion and burnout in the process of rapid educational reform [8]. Subsequently, this ultimately leads them to commit less to their schools and even quit the profession. Traditionally, the Confucian values and beliefs in China’s cultural context expect teachers to “dedicate themselves to teaching like a candle”. Such high and sometimes unrealistic expectations render teachers vulnerable to quitting. Therefore, these new challenges of educational reform and the unique low commitment profile of Chinese teachers prompt new research exploring teachers’ organizational commitment in China.

### 1.2. Leadership and Teacher Commitment

Effective school leadership is closely linked to teachers’ organizational commitment [9], with transformational, instructional and distributed leadership models being the most discussed in the literature [10]. Transformational leadership inspires teachers to exceed expectations by addressing higher-level needs, building trust and fostering organizational purpose, resulting in greater commitment and alignment with long-term goals [11]. Instructional leadership enhances commitment by focusing on teaching quality, empowering teachers to believe in their impact on student success and building their instructional capacity [12]. Distributed leadership involves collaborative decision-making with various school staff and stakeholders, leading to sustained effort, ownership and improved teacher commitment [13]. While these leadership styles emphasize external motivations and performance outcomes, authentic leadership focuses more on relational and inward aspects, potentially offering a deeper understanding of teachers’ commitment in the Chinese educational context.

First of all, unlike the three prevalent leadership theories in the realm of teacher commitment, which prioritize motivating and involving teachers in organizational goals, authentic leadership emphasizes introspection and relationship building [14]. As authentic leaders, principals exemplify positive virtues and psychological capacities, fostering trust and mutual self-development through transparent interactions and positive role modeling. Secondly, authentic leadership is viewed as a root concept or precursor to all other forms of positive leadership [15]. Meta-analytic reviews show that authentic leadership has demonstrated dominance over servant, ethical and transformational leadership when it comes to explaining organizational commitment [16,17]. Thirdly, the Chinese Confucian culture highly values moral purpose, honesty and integrity, sharing similarities with the concept of authentic leadership. Authentic leadership is a developmental form of positive leadership with the addition of ethical leadership qualities [18]. The existing related literature has shown that a moral perspective in leadership is closely related to teachers’ positive attitudes and behaviors [19].

Thus, it is important to explore how principals’ authentic leadership provides support to improve teachers’ organizational commitment in the Chinese educational context. Prior research has found that the effect of principals’ leadership on teachers’ organizational commitment is indirectly mediated by workplace-related elements [20,21], but it has not shed much light on teachers’ psychological attributes. This is obviously not sufficient for teachers who have always experienced high levels of emotional demands [22]. As noted earlier, more and more Chinese teachers are suffering from negative emotions, such as anxiety and stress. In turn, teachers’ well-being and social–emotional competence have attracted increased attention from both academia and school managers in China [23,24]. Ilies et al. [25] have confirmed that authentic leadership can significantly improve subordinates’ well-being. Based on Affective Events Theory, teachers’ well-being can affect their attitudes. Thus, teachers’ well-being was selected as a mediator in our study. Furthermore, recent research emphasizes the importance of including the boundary conditions in which principal leadership operates [26]. Teacher social–emotional competence refers to the core competence of better individual adaptation and social development by recognizing and managing one’s emotions, establishing and maintaining supportive relationships and taking responsibility for one’s decisions and actions [27]. Socially and emotionally competent teachers are sensitive enough to recognize and understand the emotions and expectations of principals and know how to manage their behavior and achieve positive goals [28]. Thus, teachers’ social–emotional competence is chosen as a moderating variable in our study.

### 1.3. Research Questions

The research questions in this study are as follows:

1. What are the effects of principals’ authentic leadership on teachers’ organizational commitment and well-being?
2. What roles do teachers’ well-being and social–emotional competence play in the relationship between principals’ authentic leadership and teachers’ organizational commitment?

## 2. Literature Review

### 2.1. Theoretical Framework

Employee emotions are increasingly recognized as pivotal in organizational dynamics, a trend echoed in educational research. Emotion accompanies, influences and shapes employees, runs through the entire work process and affects employees’ work performance. Considering these, Weiss and Cropanzano [29] proposed Affective Events Theory (AET) to explore the relationship between affective events and affective reactions among employees and their attitudes. According to AET, the work environment represents as a set of concrete or abstract features (e.g., leadership style). These features influence employees’ work attitudes through two pathways: (i) a non-emotional path, where individuals evaluate work based on standards like values and needs, and (ii) an emotional pathway, where work environment features affect specific work events (positive or negative) and then trigger emotional reactions, shaping work attitudes. Individual dispositions, such as personality traits, moderate this relationship between work environment features and individual attitudes.

This study is grounded in AET, with authentic leadership serving as a key focus due to its emphasis on genuine relationships and emotional resonance, which positively influence teachers’ commitment through trust-based interactions. First of all, unlike transformational, instructional and distributed leadership, which primarily target organizational goals, teaching quality and shared decision-making, authentic leadership caters specifically to the emotional and relational needs of teachers. By drawing from both positive psychological capacities and a well-developed organizational context [14], authentic leadership creates a supportive work environment that enhances teachers’ positive work attitudes, such as increased organizational commitment. Secondly, authentic principals are more aware of their and their teachers’ strengths and weaknesses, act in concordance with their internalized moral values, take teachers’ suggestions into consideration before making decisions and transparently share information [30]. Teachers often show high-level positive emotions in response to these events as their desired standards and needs can be met by authentic principals. The experience of happiness and satisfaction could make teachers more committed to their schools. Thirdly, teachers with high levels of social–emotional competence tend to better recognize and understand the emotions and expectations of authentic principals, colleagues and students and establish a more positive connection to the school [31]. In other words, teacher social–emotional competence, as a personality determinant, can moderate the relationship between work environment features and teachers’ attitudes.

**Figure 1:**
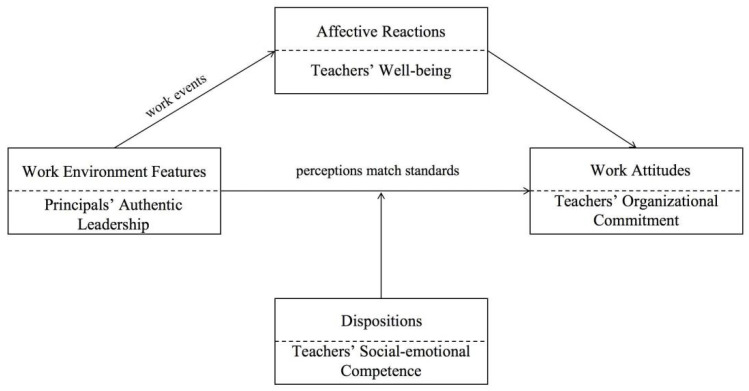
Theoretical framework derived from Affective Events Theory.

Thus, the proposed framework in this study based on AET is conceptualized in Figure 1.

### 2.2. Authentic Leadership and Teachers’ Organizational Commitment

Luthans and Avolio [14] established the cornerstone of authentic leadership theory by defining it as follows: authentic leadership is a process that leverages positive psychological capacities within a well-developed organizational context, leading to enhanced self-awareness and self-regulated positive behaviors among leaders and followers, thus facilitating positive self-development. After this, a variety of conceptual definitions and the dimensional structure of the construct have emerged. Based on a comprehensive overview of previous analyses, Walumbwa et al. [30] provided a validated dimensional structure of authentic leadership that has been dominant in research: self-awareness (i.e., knowing oneself and one’s effects on others), balanced processing (i.e., utilizing followers’ inputs in decision-making), relational transparency (i.e., exhibiting one’s authentic self without displaying fake behavior) and an internalized moral perspective (i.e., self-governing one’s behavior guided by internalized moral and non-contradictory thoughts). These key features not only distinguish authentic leaders from others, but also enable authentic leaders to promote individual and organizational flourishing. Empirical evidence has found that principals’ authentic leadership is positively associated with teacher outcomes such as teachers’ engagement and knowledge sharing [32,33].

Organizational commitment reflects teachers’ sense of loyalty to their schools and their recognition of their schools’ goals and shared values [34]. In China’s educational context, the current definition of teachers’ organizational commitment is mainly based on four aspects: affective commitment (i.e., teachers’ endorsement of school goals and values), normative commitment (i.e., teachers’ sense of obligation towards the school), ideal commitment (i.e., teachers’ consideration of whether their individual strengths can be effectively utilized within the school environment) and involvement commitment (i.e., teachers’ apprehension of the potential losses should they leave their current position) [35]. It is important to understand the process through which teachers commit to their schools because organizational commitment is the psychological bond that maintains the relationship between teachers and schools [36].

Our choice of authentic leadership as the antecedent of teachers’ organizational commitment is informed by four core components of authentic leadership. First, authentic leaders exhibit heightened self-awareness, fostering genuine interactions that enhance teachers’ trust and personal identity, thus bolstering their commitment to the school [37]. Secondly, balanced processing enables authentic principals to make objective decisions and solicit input, empowering teachers to realize their potential within the organization. Thirdly, internalized moral perspectives guide authentic leaders’ consistent ethical behavior, earning teachers’ appreciation and fostering organizational loyalty [38]. Finally, relational transparency fosters trust and motivation among teachers, diminishing intentions to leave [39]. Indeed, previous studies have consistently demonstrated positive relationships between authentic leadership and employees’ organizational commitment across various sectors, including hospitality, nursing and tourism agencies [40,41,42].

Hence, we suppose the following.

**Hypothesis 1:**  Principals’ authentic leadership has positive effects on teachers’ organizational commitment.

### 2.3. The Mediating Role of Teachers’ Well-Being 

Authentic leadership is an important contextual stimulus for teachers’ organizational commitment, and there must be an internal drive to enable commitment. Teachers’ well-being encompasses their feelings regarding job satisfaction and life satisfaction, as well as their psychological experiences in both professional and personal spheres [43]. This constitutes a crucial intrinsic psychological asset. Previous studies have confirmed that employee well-being is a crucial predictor of organizational commitment [44,45]. For example, an empirical study in the South Asian context found that subjective well-being positively and indirectly influenced the professional commitment of nurses and doctors [46]. Similarly, research in China’s educational context has demonstrated that kindergarten teachers’ occupational well-being positively predicts their occupational commitment [47]. Teachers with high-level well-being have a strong emotional attachment and sense of responsibility to the school [48]. In this sense, teachers’ well-being can exert a positive influence on their affective commitment and normative commitment. In addition, well-being is a driving force for teachers’ individual growth and development, which may promote teachers to pursue and realize their career ideals, i.e., teachers’ well-being has a positive effect on their ideal commitment. Finally, teachers perceive their well-being as the benefit of continuing to work in school [49], which in turn fosters their willingness to dedicate their efforts to their work. Therefore, teachers’ well-being can promote their involvement and commitment.

Based on the above analysis, we suppose the following.

**Hypothesis 2:**  Teachers’ well-being has positive effects on teachers’ organizational commitment.

Authentic leadership, as a typical positive leadership style that originates from and promotes positive mental abilities and a moral atmosphere [30], can stimulate employees’ positive emotions and improve their psychological strength. Employees’ good emotional status reflects their well-being [50], and positive psychological power can affect their self-regulation, so that they can better adjust to work and life, leading to improved well-being. The theorists on authentic leadership indicate that leader authenticity can impact and benefit followers’ well-being through enhancing their authenticity [51]. Authentic principals frequently provide opportunities for teachers to express their thoughts and perspectives, actively involving them in decision-making and goal-setting processes. Teachers may feel empowered and included, recognizing that their efforts are valued. In a democratic environment fostered by authentic leadership, teachers are likely to experience lower levels of stress and burnout, along with higher levels of well-being [52]. Prior empirical studies also position authentic leadership as an important antecedent of employees’ well-being. Brunetto et al. [53] found that authentic leadership promoted employees’ well-being in terms of Conservation of Resources Theory when surveying Australian not-for-profit employees. From a social psychology perspective, Lopez and Rice argued that the qualities of authentic leadership can provide employees with a sense of security, reduce uncertainty and decrease the frequency of role transitions, thereby enhancing their well-being [54]. In educational settings, Shie and Chang’s analysis of Taiwanese senior high/vocational school teachers also found that authentic leadership could positively predict teachers’ well-being [55].

In this way, we suppose the following.

**Hypothesis 3:**  Principal authentic leadership has positive effects on teachers’ well-being.

Building on Hypotheses 2 and 3, we propose that principals’ authentic leadership not only directly influences teachers’ organizational commitment but also that teachers’ well-being may serve as a crucial mediating factor. While empirical research on this specific pathway remains limited, related studies provide support for this proposition. For instance, research involving 428 employees found that empowering leadership positively impacts affective commitment through the partial mediation of well-being [56]. Similarly, a study with professionals from Indian public sector banks highlighted the mediating effect of psychological well-being on the relationship between transformational leadership and organizational commitment [57]. Furthermore, previous research has demonstrated the mediating role of teachers’ well-being in linking principal transformational leadership with teachers’ workplace attitudes and reducing burnout [58]. According to Affective Events Theory, authentic principals seek feedback to improve their interactions with teachers, genuinely present themselves to followers and exhibit sincerity in their behaviors, whereby teachers perceive that they are placed as a priority and their positive emotional experiences are increased through these authentic events. In turn, teachers may form positive emotional connections to the principal and the school [29]. Teachers’ well-being thereby serves as a key mediating link between authentic leadership and teachers’ organizational commitment.

Thus, we suppose the following.

**Hypothesis 4:**  Teachers’ well-being mediates the relationship between authentic leadership and teachers’ organizational commitment.

### 2.4. The Moderating Role of Social–Emotional Competence

Social–emotional competence, derived from emotional intelligence, refers to the ability to recognize and manage one’s emotions and relationships with oneself and others, which is developed in the complex process of growth and development [59]. Emotional intelligence (EI) is considered an individual difference variable that moderates the relationship between external stimuli and behavioral outcomes. Individuals with varying levels of EI exhibit different cognitive responses and emotional regulation abilities when confronted with environmental stimuli, leading to distinct behaviors and results. A longitudinal study indicated that EI moderates the relationship between job insecurity and physical complaints [60], suggesting that, during times of job uncertainty, employees’ ability to manage their emotions and maintain relationships with their supervisors is a critical resource in safeguarding employee outcomes. Another study confirmed that EI positively and significantly moderates the relationship between internet usage and impulsive buying behavior [61]. Furthermore, research has shown that understanding the moderating effects of EI on the relationships among high-involvement human resource management practices, affective commitment and flow is crucial [62]. Therefore, in this study, teachers’ social–emotional competence may also play a moderating role between principals’ authentic leadership and teachers’ organizational commitment. According to Affective Events Theory, teachers with a high level of social–emotional competence are more flexible in interacting with others and tend to better recognize and understand the emotions and expectations of authentic principals, leading to positive connections to the school [31]. In contrast, teachers who lack social–emotional competence have a weaker understanding of principals’ emotions, goals and values and may not be able to respond well, thus weakening the effectiveness of authentic leadership. A meta-analytic study showed that individual’s emotional intelligence is positively associated with his/her career commitment [63].

Based on the above analysis, we suppose the following.

**Hypothesis 5:**  Teachers’ social–emotional competence moderates the relationship between principals’ authentic leadership and teachers’ organizational commitment, such that this relationship is stronger among teachers with a high level of social–emotional competence than those with a low level.

## 3. Research Methodology 

### 3.1. Data Collection and Participants

The data for this study came from a district in Beijing, renowned for its abundant high-quality educational resources and skilled teaching staff. However, teachers in this area face heightened professional pressure and societal expectations. Particularly notable in the competitive district of Haidian, primary schools experience the pervasive “chicken parenting” phenomenon, placing unprecedented demands on both students and teachers. Moreover, as China’s capital and a key center for cultural and educational activities, Beijing often serves as a trendsetter for national educational policies. Exploring teachers’ organizational commitment in this context can illuminate crucial aspects such as frontline teachers’ sense of belonging, dedication and affiliation with their schools amidst ongoing educational reforms. This research carries significant implications in terms of enhancing the stability of teachers in other regions across China. Therefore, Beijing was selected for this study.

Convenience and purposive sampling methods were employed in this study. First, with the assistance of the local education authorities in Beijing, we established contact with 30 primary and secondary schools, randomly selected from across the city. During the initial outreach, we introduced the purpose, significance and participation requirements of the study to each school and sought feedback from school management. Based on factors such as the school development level, geographic location and management input, we ultimately selected seven primary schools to distribute the questionnaire. The questionnaire included items on four main variables and some demographic information. At the beginning of the questionnaire, participants were informed about the anonymity of the study and how their responses would be protected. Teachers from these seven schools participated voluntarily and anonymously, providing informed consent. The questionnaire took approximately 15 min to complete, after which participants could submit their responses.

In total, 600 teachers participated in our survey, among which 476 valid responses were collected, representing a response rate of 79.33%. The majority of the respondents (85.92%) were females and 14.08% were males. Most teachers held a bachelor’s degree, comprising 86.55% of the respondents. The mean age of the teachers was approximately 37.82 and the average number of years of teaching experience was 15.76. In addition, over half of the teachers served as class teachers, accounting for 53.78%. These characteristics of the overall teacher sample reflect the national distribution of primary school teachers in China. Detailed demographic information is presented in Table 1.

### 3.2. Instruments and Measures

According to the conceptualized theoretical framework, there are four main scales in the hypothetical model, as shown in Figure 1. A five-point Likert-type scale ranging from 1 (strongly disagree) to 5 (strongly agree) was used to measure teachers’ perceptions of authentic leadership, teachers’ organizational commitment and their social–emotional competence, whereas teachers’ well-being was measured on a 7-point scale between 1 (strongly disagree) and 7 (strongly agree). The obtained data from the main study were used to test the validity and reliability of the instrument, as well as seeking evidence to examine the relationships among the scales in the hypothetical model.

According to the results of the confirmatory factor analysis (CFA), items with a standardized factor load of less than 0.4 in each of the scales in the measurement model were eliminated, ensuring that the questionnaire was suitable for this study [64]. For the composite reliability (CR) value, we adopted the criterion recommended by Fornell and Larcker [65], i.e., Cronbach’s α should be higher than 0.7. For the structural validity, Wen et al. [66] suggest that the χ^2^/*df,* root mean square error of approximation (RMSEA) and standardized root mean square residual (SRMR) should usually be set at less than 10, 0.1 and 0.1, respectively. The bottom-line standard for the comparative fit index (CFI) and Tucker–Lewis index (TLI) was 0.9. Then, the reliability and validity of the four scales were analyzed. Table 2 presents the CFA results and alpha coefficients of the four scales used in the current study.

Authentic leadership. With reference to the work by Walumbwa et al. [30], a sixteen-item scale was developed to measure teachers’ perceptions of principals’ authentic leadership. The scale consisted of four dimensions: self-awareness (four items), internalized moral perspective (four items), balanced processing (three items) and relational transparency (five items). Sample items included “My principal understands the potential impact of certain actions on others”, “My principal demonstrates beliefs that are consistent with actions”, “My principal listens carefully to different points of view before coming to conclusions” and “My principal is willing to reveal his/her true feelings to us”. The Cronbach’s alpha was 0.968 in this study, indicating good reliability. The CFA results showed that χ2/df = 5.062, RMSEA = 0.092, SRMR = 0.022, CFI = 0.959 and TLI= 0.949, indicating that the validity was acceptable.

Teachers’ organizational commitment. With reference to the work by Song and Cai [67], a fourteen-item scale was developed to measure teachers’ organizational commitment. The scale consisted of four dimensions: affective commitment (five items), normative commitment (three items), ideal commitment (three items) and involvement commitment (three items). With the exception of the reverse scoring method used for one item, the higher the score for the other items, the higher the level of teachers’ organizational commitment. The exception item was “I think working in this school is just to earn money to support the family”. The other sample items included “I find that my values align very closely with the values of the school”, “Since I can achieve my ideals and aspirations at my current school, I am willing to stay and work hard” and “If I were to leave my current school, I would lose many benefits and perks”. The Cronbach’s alpha was 0.885 in this study, indicating good reliability. The CFA results showed that χ2/df = 4.577, RMSEA = 0.087, SRMR = 0.055, CFI = 0.921 and TLI = 0.901, indicating that the validity was acceptable.

Teachers’ well-being. With reference to the work by Zheng et al. [43], an eighteen-item scale was developed to measure teachers’ well-being. The scale contained the dimensions of life well-being, workplace well-being and psychological well-being, with each dimension consisting of six items, examples of which include “I am in a good life situation”, “I feel basically satisfied with my work achievements in my current job” and “I feel positive about myself and am confident in my abilities”. The Cronbach’s alpha was 0.955 in this study, demonstrating high internal consistency. The CFA yielded satisfactory results, indicating good structural and model fitting validity: χ2/df = 5.108, RMSEA = 0.093, SRMR = 0.042, CFI = 0.931, TLI = 0.921.

Teachers’ social–emotional competence. With reference to the work by the UNICEF Social Emotional Learning Project Team and Li et al. [68], a 22-item scale was developed to measure teachers’ social–emotional competence, which contained six dimensions: self-awareness (four items), self-management (four items), others’ awareness (four items), others’ management (three items), collective awareness (three items) and collective management (four items). Sample items included “I know what affects my mood”, “I can resolve conflicts with others through communication” and “I play my role effectively within the team and take on corresponding responsibilities”. The Cronbach’s alpha was 0.943 in this study, demonstrating high internal consistency. The CFA yielded satisfactory results, indicating good structural and model fitting validity: χ2/df = 3.570, RMSEA = 0.074, SRMR = 0.053, CFI = 0.916, TLI = 0.903.

Control variables. Considering the possible influence of teachers’ demographic variables on teachers’ organizational commitment, we included teachers’ gender, age, education, position and teaching experience as the control variables in the present study.

### 3.3. Data Analyses

All analyses were executed using SPSS 26.0 and AMOS 21.0. We conducted confirmatory factor analysis (CFA) to ensure the distinctiveness of the four variables in this study. Table 3 presents the results of the discriminant validity analysis. The bold number is the square root value of the average variance extracted (AVE). If the square root of the AVE value is greater than the correlation coefficient between the variable and other variables, it indicates that the four variables have good discriminant validity [65]. As shown in Table 3, the AVE square root value of each variable is greater than the absolute value of the correlation coefficient between that variable and the other variables, providing evidence for the constructs’ distinction between authentic leadership, teachers’ organizational commitment, teachers’ well-being and social–emotional competence.

Basic descriptive statistical tests and a correlation analysis were conducted using SPSS 26.0. As shown in Table 3, authentic leadership was positively correlated with teachers’ organizational commitment (*r* = 0.598, *p* < 0.01) and teachers’ well-being (*r* = 0.432, *p* < 0.01), and teachers’ organizational commitment was positively correlated with teachers’ well-being (*r* = 0.589, *p* < 0.01). Additionally, social–emotional competence was positively related to authentic leadership (*r* = 0.483, *p* < 0.01), teachers’ well-being (*r* = 0.485, *p* < 0.01) and teachers’ organizational commitment (*r* = 0.503, *p* < 0.01). The correlations among most of the variables were in the expected direction, providing support for further hypothesis testing.

Structural equation modeling (SEM), regression analyses and bootstrapping tests were conducted, following the recommendations of Preacher and Hayes [69], to test the hypotheses. SEM was used to test the relationships among the variables of authentic leadership, teachers’ well-being and teachers’ organizational commitment. Hierarchical regression analyses were conducted to examine the moderating role of teachers’ social–emotional competence. The bootstrap method was used to test the mediating and moderating effects.

## 4. Results

### 4.1. Testing the Main Effect

**Figure 2:**
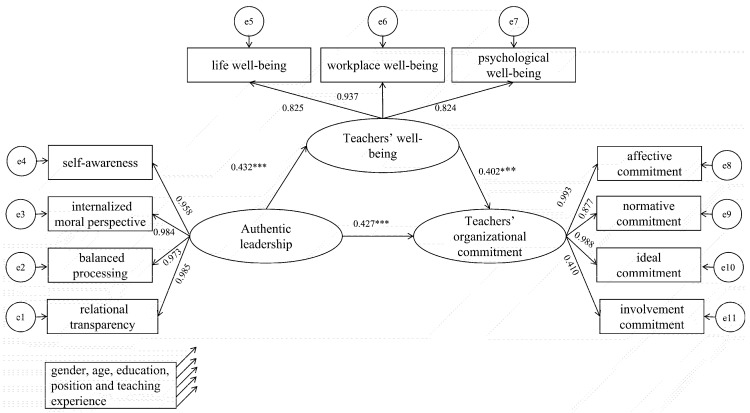
Structural equation modeling (SEM) of authentic leadership, teachers’ well-being and teachers’ organizational commitment. Note: *N* = 476, *** *p* < 0.001.

Figure 2 presents the SEM of authentic leadership and teachers’ organizational commitment, with gender, age, education, position and teaching experience as control variables. Authentic leadership was set as the independent variable, teachers’ organizational commitment as the dependent variable and teachers’ well-being as the mediating variable. The model fit indices (χ2/df = 2.896, RMSEA = 0.063, SRMR = 0.050, CFI = 0.908, TLI = 0.901) were within the suggested values to further explain the model.

As depicted in Figure 2, authentic leadership was a significant positive predictor of teachers’ organizational commitment (β = 0.427, *p* < 0.001), providing support for H1. Teachers’ well-being displayed a significant positive predictive effect on teachers’ organizational commitment (*β* = 0.402, *p* < 0.001), supporting H2. Furthermore, authentic leadership exhibited a significant positive predictive effect on teachers’ well-being (*β* = 0.432, *p* < 0.001), lending support to H3.

### 4.2. Testing the Mediated Effects Model

To examine whether teachers’ well-being acted as a mediator between authentic leadership and teachers’ organizational commitment (Hypothesis 4), a bootstrapping approach with the aid of the SPSS macro was used [70]. We estimated the 95% bias-corrected confidence intervals (CIs) for indirect effects with 5000 bootstrapped re-samples. The indirect effect is significant when the bias-corrected 95% CI does not include zero. The results showed that the indirect effect of authentic leadership on teachers’ organizational commitment through teachers’ well-being amounted to 0.174 (95% bias-corrected CI [0.132, 0.219]), which suggests that the relationship between authentic leadership and teachers’ organizational commitment is partially mediated by teachers’ well-being. Thus, Hypothesis 4 is supported.

### 4.3. Testing the Moderated Effects Model

**Figure 3:**
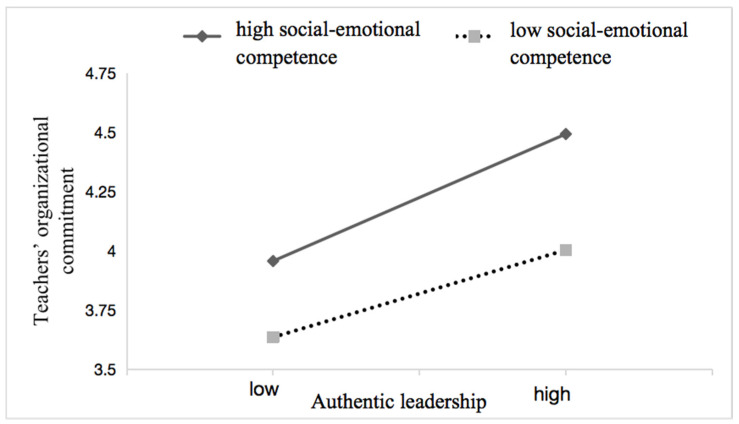
Moderating effect of social–emotional competence on authentic leadership and teachers’ organizational commitment.

To test Hypothesis 5, we adopted Model 5 from the SPSS macro developed by Preacher et al. [70]. When undertaking the moderation analysis, we centered the variables in the interaction term at the mean. If the interaction term (independent variable × moderator variable) is significant, it indicates a moderating effect. As shown in Table 4, the coefficient of the interaction term between authentic leadership and social–emotional competence on teachers’ organizational commitment was significant (*β* = 0.089, *p* = 0.013 < 0.05). Additionally, we plotted a simple slope to visually represent how the moderating variable (social–emotional competence) influenced the relationship between the independent variable (authentic leadership) and the dependent variable (teachers’ organizational commitment) at different levels. As shown in Figure 3, teachers with high social–emotional competence (one standard deviation above the mean) exhibited a stronger relationship between authentic leadership and teachers’ organizational commitment than teachers with low social–emotional competence (one standard deviation below the mean). This supports Hypothesis 5, which posits that social–emotional competence moderates the relationship between authentic leadership and teachers’ organizational commitment.

## 5. Discussion

Given that authentic leadership has been found to be a powerful predictor of teachers’ valued workplace attitudes and behaviors, a better understanding of how and under what boundary conditions authentic leadership contributes to these outcomes is very important [71]. In this investigation, in the Chinese school context, we constructed a model with teachers’ well-being as a mediating variable and social–emotional competence as a moderating variable to further clarify how principals’ authentic leadership influences teachers’ organizational commitment. Compared with previous studies, the present study on Chinese sampled schools differs in the following two ways. First, the study was explicitly based on Affective Events Theory to explore the relationship between authentic leadership and teachers’ organizational commitment, further expanding the application of Affective Events Theory in educational settings and deepening the understanding of authentic leadership’s effectiveness in China’s school context. Second, we developed a moderated mediation framework by incorporating teachers’ well-being as a mediator and social–emotional competence as a moderator, emphasizing the specific role of teacher affective variables in promoting teachers’ organizational commitment. Below, we further interpret the findings, elaborate the practical implications and examine the limitations of this study.

### 5.1. Interpretation of the Findings

First, we found a significant and positive impact of authentic leadership on teachers’ organizational commitment in the sampled Chinese schools. This suggest that teachers who admire the principal’s integrity tend to be more committed to their school. In contrast, teachers will lose trust in the school if the principal exhibits inauthentic behaviors [72], thereby leading to teacher turnover. This finding is consistent with previous studies. For example, Baek et al. [41] identified the contribution of authentic leadership to employees’ organizational commitment using survey data from 1118 nurses in Korea. Our study further provides empirical evidence of the effectiveness of authentic leadership and explains the relationship between authentic leadership and teachers’ organizational commitment in China’s educational setting, which answers the call for the examination of leadership effectiveness in different cultural and educational contexts [73]. In the context of China’s principal responsibility system and the Confucian emphasis on “guanxi” (interpersonal relationships) and moral conduct [74], principals are regarded as key authority figures, making teachers more likely to consciously or unconsciously emulate their authentic behaviors [75]. This emulation fosters shared values and enhances their commitment to the school organization [35]. While the existing literature extensively links transformational, instructional and distributed leadership with teacher commitment, the impact of authentic leadership remains underexplored. Our study fills this gap by demonstrating that leadership styles emphasizing genuine relationships and ethical behavior can significantly enhance teachers’ affective, normative, ideal and involvement commitment. This finding offers valuable insights into how fostering authenticity in principal leadership can help to build a stable and effective teaching workforce.

Secondly, our study has also confirmed that authentic leadership has an indirect effect on teachers’ organizational commitment through the mediating role of teachers’ well-being, which aligns with the perspectives expressed by Affective Events Theory. Specifically, this finding suggests that authentic principals, who are often confident, optimistic about the future, resilient, morally grounded and actively foster these qualities in teachers, encourage teachers’ self-determination and psychological engagement [76]. These leadership behaviors translate into specific work events that create a sense of joy and support for teachers, simultaneously promoting their positive growth and self-development [77]. As a result, teachers become better at recognizing and regulating their emotions, which boosts their well-being [78]. When teachers experience enhanced well-being, they develop strong emotional bonds with their school, align closely with its organizational goals and show a heightened commitment to remaining part of the institution [48]. This process exemplifies the emotional pathway outlined in Affective Events Theory, whereby authentic leadership fosters positive work events, which in turn trigger positive emotional responses and cultivate constructive work attitudes. In addition, previous studies have pointed out that principal authentic leadership can improve teachers’ well-being [79], and teachers’ well-being is positively related to their occupational commitment [47]. Our study further reveals the mechanism between principals’ authentic leadership and teachers’ organizational commitment by incorporating teachers’ well-being as a mediator. This is in line with the model of authentic leader and follower development proposed by Gardner et al. [80], which suggests that authentic leaders promote followers’ well-being by modeling, encouraging and developing authenticity, thereby leading to veritable and sustainable follower performance. By empirically validating that principals’ authentic leadership positively impacts teachers’ organizational commitment through the mediation of teachers’ well-being, our study enriches the concept of follower performance within this model and provides additional empirical support for this theoretical framework in the educational context.

Last, our research reveals that social–emotional competence moderates the relationship between authentic leadership and teachers’ organizational commitment, answering the call for the inclusion of more personal variables as moderators to show the effect of authentic leadership on followers’ outcomes [81]. This finding suggests that the effect of authentic leadership on teachers’ organizational commitment is stronger when teachers exhibit higher levels of social–emotional competence. This discovery clarifies the boundary conditions of authentic leadership on organizational commitment, emphasizing the pivotal role of teachers’ social–emotional competence, which aligns with the importance of individual dispositions as discussed in Affective Events Theory. Specifically, social–emotional competence is crucial in enhancing individual adaptability and social development [82]. Teachers with high social–emotional competence are self-aware, resilient, proactive, good listeners and can better understand others’ feelings and experiences [83]. They exhibit a strong sense of community and prosocial behavior, making them more responsive to principals who transparently share information, set ethical examples and involve followers in decision-making. This responsiveness leads to greater emotional resonance with effective authentic leadership behaviors, thereby deepening their sense of belonging and loyalty to the organization. However, few existing studies have examined the effects of teachers’ social–emotional competence on organizational commitment in Chinese schools. Some scholars have pointed out that social–emotional competence is a necessary professional competence for teachers in the 21st century and recommended the performance of more contextualized and grounded research in this area [84]. Responding to this call, our research has provided empirical evidence for the moderating role of social–emotional competence and contributed to understanding how teachers’ social–emotional competence moderates the relationship between principals’ leadership and teachers’ outcomes in China’s educational setting.

### 5.2. Practical Implications

First, the findings of our study highlight the importance of a focus on principals’ authenticity, which is aligned with the traditional Chinese culture. Influenced by the Confucian tradition, leaders are expected to be fair, honest and trustworthy and embody moral principles [85]. To enhance teachers’ organizational commitment, principals should demonstrate authentic leadership in several ways. Primarily, regular self-reflection is crucial for principals to improve their self-awareness. In China’s centralized education system, principals sometimes prioritize administrative tasks over understanding their own strengths and the needs of their teachers [75]. By participating in self-development training, principals can better understand themselves, effectively connect with their staff and positively influence teachers’ emotions and work attitudes. Additionally, principals should embody high moral standards, serving as ethical role models. When teachers observe principals consistently demonstrating integrity and fairness, they are more likely to emulate these behaviors, adhere to school policies and engage in school activities. Furthermore, principals should foster an open and inclusive environment that encourages teachers to voice diverse opinions. An effective information flow through regular faculty meetings and discussion forums helps to address potential issues and reduces uncertainty, enhancing teachers’ sense of security and job satisfaction. Lastly, building relationships characterized by openness and transparency is vital. By promoting mutual respect and support, principals can cultivate a collaborative and trusting work environment. When teachers feel respected and valued, they are more likely to develop a strong emotional bond with the school, contributing to their long-term commitment and reducing the likelihood of voluntary turnover. Similarly, it is important to pay attention to these attributes in the selection of principals for schools and to incorporate the theory and practice of authentic leadership into professional development programs for school principals. 

Secondly, our study indicates that teachers’ well-being plays a significant mediating role in the relationship between authentic leadership and teachers’ organizational commitment, which is a step forward in uncovering the psychological mechanism through which principal leadership affects teachers’ workplace attitudes. Previous studies have confirmed that teachers’ well-being comprises life well-being, workplace well-being and psychological well-being [58], suggesting that school leaders should show more genuine concern for teachers’ lives and psychological well-being, including their work–life balance, family happiness, emotional health, personal growth and environmental mastery, instead of just focusing on teachers’ job satisfaction [43]. Recognizing that a healthy work-life balance is crucial to maintaining high levels of well-being [86], school leaders can implement flexible work arrangements, such as flexible hours and remote work options, to help teachers to better balance their professional and personal lives. Encouraging teachers to schedule regular time off for rest and relaxation can also help to prevent burnout. Given the close link between teachers’ family well-being and their job performance [87], school leaders should support teachers’ family lives. This might include establishing support groups and offering marriage counseling, parenting advice and other services to help teachers to navigate personal issues. Additionally, providing psychological counseling services can help to mitigate the negative effects of work-related stress. To enhance psychological well-being, school leaders should strive to create a supportive work environment, which includes providing adequate resources, ensuring fair evaluation systems and fostering positive interpersonal relationships. Team-building activities that increase the mutual understanding and trust among colleagues can effectively promote a collaborative spirit and create a positive work atmosphere. In short, the findings of this study can help school leaders to better understand teachers’ overall well-being and take necessary actions to improve it, which in turn can boost teachers’ positive work-related outcomes, including teachers’ organizational commitment.

Thirdly, our study highlights the significance of social–emotional competence. Teachers’ social–emotional competence is not only a necessary professional quality for teachers themselves to cope with the complexity of the workplace and the daily interactions within the classroom, but also a significant capability in nurturing children’s psychological outcomes [28]. However, many principals only focus on developing teachers’ instructional abilities and neglect teachers’ social–emotional competence. Therefore, there is an imperative need for school leaders to take a variety of strategies and measures to develop teachers’ social–emotional competence. Initially, principals should implement social–emotional training programs, such as the “CARE” (Cultivating Awareness and Resilience in Education) program from the Garrison Institute [88]. These programs cover emotional skills, mindfulness, stress relief and interpersonal skills, helping teachers to improve their self-awareness, emotional regulation and empathy. Subsequently, fostering a positive, inclusive and open school culture is essential for the effective implementation of social–emotional learning [89]. This can be achieved through team-building activities, recognizing teacher achievements and promoting collaboration across departments. A nurturing environment increases teachers’ well-being and commitment to their schools. Lastly, enhancing family–school collaboration and community engagement is crucial in developing social–emotional competence [90]. Schools should strengthen their ties with parents and community members through meetings and events, enabling teachers to apply their social–emotional skills in broader contexts. 

## 6. Limitations and Future Research

Despite its contributions, this study faces limitations. Its cross-sectional design limits the ability to infer causality among the studied constructs. Future research should utilize longitudinal or experimental designs for more definitive causal conclusions. The focus on a small sample of Chinese primary schools also constrains the generalizability of the findings, suggesting the need for replication across diverse educational settings and larger samples. Additionally, given the nested nature of the data collected from the school environment (i.e., teachers nested within schools), it is necessary to address multilevel issues in the analysis. However, due to the insufficient number of sample groups, which did not allow for a reliable cross-level mediation and moderation analysis [91], we did not conduct a multilevel analysis in this study, representing a limitation. We strongly recommend that future researchers employ multilevel structural equation modeling to test the cross-level mediation and moderation effects [92]. Lastly, while this research incorporated teachers’ well-being as a mediator and social–emotional competence as a moderator to shed light on the psychological mechanism and boundary conditions of authentic leadership on teachers’ organizational commitment, further studies should investigate other relevant organizational and personal factors, such as teachers’ professionalization and social status, teachers’ expectations of students and beliefs about student learning, teacher burnout and teacher resilience, professional learning communities and so forth.

## 7. Conclusions

Based on Affective Events Theory, this research enhances our empirical understanding of how and under what conditions principals’ authentic leadership contributes to teachers’ organizational commitment. This is of great importance given the global issue of high teacher attrition rates and the subsequent shortage of high-quality teachers. Our findings suggest that principals’ authentic leadership positively influences teachers’ organizational commitment. Additionally, our study confirms that principals’ authentic leadership indirectly affects teachers’ organizational commitment through the mediating role of teachers’ well-being. Moreover, the effect of authentic leadership on organizational commitment is stronger when teachers’ social–emotional competence is higher. Therefore, our study underscores the importance of enhancing principals’ authenticity and integrity; focusing on teachers’ life, workplace and psychological well-being; and fostering teachers’ social–emotional competence.

## Figures and Tables

**Table 1:** Demographic characteristics of the sample (*N* = 476).

Characteristics	*N*	%
Gender		
Male	67	14.08
Female	409	85.92
Position		
Class teacher	256	53.78
Regular teacher	220	46.22
Educational qualifications		
High school diploma	12	2.52
Bachelor’s degree	412	86.55
Master’s degree and above	52	10.92

**Table 2:** CFA results and alpha coefficients of the scales.

Scale	No. of Items	χ^2^/*df*	RMSEA	SRMR	CFI	TLI	Cronbach’s Alpha
Authentic Leadership	16	5.062	0.092	0.022	0.959	0.949	0.968
Teachers’ Organizational Commitment	14	4.577	0.087	0.055	0.921	0.901	0.885
Teachers’ Well-Being	18	5.108	0.093	0.042	0.931	0.921	0.955
Teachers’ Social–Emotional Competence	22	3.570	0.074	0.053	0.916	0.903	0.943

RMSEA: root mean square error of approximation; SRMR: standardized root mean square residual; CFI: comparative fit index; TLI: Tucker–Lewis index.

**Table 3:** Discriminant validity, descriptive and correlation analysis.

	M	SD	1	2	3	4
1. Teachers’ well-being	5.528	0.838	**0.755**			
2. Social–emotional competence	4.259	0.477	0.485 ***	**0.671**		
3. Authentic leadership	4.332	0.660	0.432 ***	0.483 ***	**0.837**	
4. Organizational commitment	3.727	0.604	0.589 ***	0.503 ***	0.598 ***	**0.647**

Notes: *** *p* < 0.001 (two-tailed); M: mean; SD: standard deviation. The bold number is the square root value of the AVE.

**Table 4:** Results of the moderation analysis.

	Teachers’ Organizational Commitment			
	*SE*	*t*	*p*	*β*
Control variables				
Gender	0.062	−0.608	0.543	−0.022
Class teacher	0.044	1.553	0.121	0.057
Age	0.056	1.987	0.048 *	0.144
Education	0.062	−1.587	0.113	−0.058
Teaching experience	0.024	−0.784	0.433	−0.057
Moderating effect				
Authentic leadership × Social–emotional competence	0.070	2.497	0.013 *	0.089
*R* ^2^	0.443			
Adjusted *R*^2^	0.434			

* *p* < 0.05.

## Data Availability

The data that support the findings of this study are available from the corresponding author upon reasonable request.
